# Relationship between Age at Diagnosis of ADHD and ASD

**DOI:** 10.15844/pedneurbriefs-29-10-5

**Published:** 2015-10

**Authors:** Michelle M. Yee, J. Gordon Millichap

**Affiliations:** 1 Division of Neurology, Ann & Robert H. Lurie Children's Hospital of Chicago, Chicago, IL; 2 Departments of Pediatrics and Neurology, Northwestern University Feinberg School of Medicine, Chicago, IL

**Keywords:** ADHD, Autism, DSM-IV-TR, DSM-5

## Abstract

Investigators from the Division of Developmental Medicine and Clinical Research Center, Boston Children's Hospital and Harvard Medical School, Boston, Massachusetts, studied the relationship between the timing of Attention Deficit Hyperactivity Disorder (ADHD) diagnosis in children with Autism Spectrum Disorder (ASD) and the age at ASD diagnosis.

Investigators from the Division of Developmental Medicine and Clinical Research Center, Boston Children's Hospital and Harvard Medical School, Boston, Massachusetts, studied the relationship between the timing of Attention Deficit Hyperactivity Disorder (ADHD) diagnosis in children with Autism Spectrum Disorder (ASD) and the age at ASD diagnosis. Prior studies have suggested that symptoms of ADHD may overshadow or mask the symptoms of ASD but none has examined the relationship between the age at ADHD diagnosis and the age at ASD diagnosis. Data were drawn from the 2011-2012 National Survey of Children's Health, which asked parents to provide the age at which their child received a diagnosis of ADHD and/or ASD. There were 1496 children with a current diagnosis of ASD reported by parents of children between ages 2 to 17 years; approximately 20% of these children had been initially diagnosed with ADHD. Children diagnosed with ADHD before ASD were diagnosed with ASD ~3 years (95% confidence interval 2.3-3.5) after children in whom ADHD was diagnosed at the same time or after ASD. The children with ADHD diagnosed first were 30 times more likely to receive their ASD diagnosis after age 6 (95% confidence interval 11.2-77.8). The disparity in delay of age for ASD diagnosis for the ADHD before ASD group was maintained across all severity levels. The findings support implications that children with ADHD before ASD may exhibit unique dimensional traits that could bias clinicians toward an ADHD diagnosis. Diagnostic criteria and screening measures for ASD may need to reflect the overlapping symptomatology between ASD and ADHD. [1]

COMMENTARY. The new DSM-5 restructuring of the ASD and ADHD diagnostic categories has caused concern about how these changes may impact prevalence rates, and rates of comorbid psychopathology. Under the DSM-5, symptoms of ADHD should be present before age 12, not 7 years, and the number of symptoms required is 5, not 6. To investigate the prevalence of inattention and impulsive symptoms, 1722 infants and toddlers were separated into three diagnostic groups for analyses: DSM-5 ASD group, an atypically developing group, and a DSM-IV-TR ASD group. Significantly elevated rates of inattention/impulsive symptoms were identified in toddlers meeting DSM-5 criteria for ASD. ASD symptom severity was positively correlated with inattention/impulsive symptoms regardless of the primary diagnosis. The expression of impulsive and inattentive symptoms did not differ significantly within diagnostic groups [2]. The similarity of symptoms is supportive of the theory that ASD and ADHD represent a continuum, a deviance from an acceptable norm, and having a common origin [3]. ASD prevalence estimates may be lower under DSM-5 criteria [4], but the need for treatment is dependent on multiple factors, and not restricted to an arbitrary number of DSM criteria.
